# Effects of periodontal treatment on periodontal status in Finland: a register-based study

**DOI:** 10.2340/aos.v84.43232

**Published:** 2025-03-18

**Authors:** Anna Haukka, Minna Kaila, Jari Haukka, Anna Maria Heikkinen

**Affiliations:** a Social Services, Health Care and Rescue Services Division, City of Helsinki, Helsinki, Finland; b Department of Public Health, University of Helsinki, Helsinki, Finland; c Department of General Practice and Primary Health Care, University of Helsinki, Helsinki, Finland; d Public Health Medicine, Department of Public Health, University of Helsinki, Helsinki, Finland; e Faculty of Medicine and Health Technology, University of Tampere, Tampere, Finland

**Keywords:** Oral health, periodontal treatment, mental disorders, diabetes mellitus, cardiovascular disease

## Abstract

**Objective:**

This register-based follow-up study investigated periodontal status after periodontal treatment (PT) based on need following oral health examination (OHE).

**Materials and methods:**

A total of 42,533 adults aged 18–89 years receiving OHE in the public oral health clinics of the City of Helsinki in 2009 were included. Dentists recorded periodontal status by the Community Periodontal Index (CPI), and determined the individual recall interval (IRI). Follow-up OHE between 2010 and 2015 was performed for 16,040 adults based on IRI or later. Outcome of interest was change of CPI during follow-up and was modelled with proportional odds model for each sextant separately. Results were reported as odds ratios (ORs).

**Results:**

Signs of periodontal disease were present in 95% of the study population. Symptoms of periodontitis (CPI score 3 or 4) were observed in 24% of patients. In models, PT indicated better outcome in all six sextants and in sextant 5 after one treatment (OR 5.05, 95% confidence interval [CI] 4.53–5.63). A poorer outcome was observed in patients with diabetes or severe mental disorders and in men.

**Conclusions:**

The study population had a high prevalence of periodontal diseases. Men and patients with diabetes or severe mental disorders should be specifically targeted by dentists.

## Introduction

Oral health care services primarily aim to prevent oral diseases and identify progression of caries, periodontal diseases, and mucosal changes [1]. As caries prevalence continues to decrease in developed countries (such as Finland), periodontal diseases, such as gingivitis and periodontitis, are the most prevalent oral diseases and are a burden to adult oral health worldwide [2–4].

Periodontal diseases include numerous biofilm-initiated conditions with inflammatory responses. The cause of gingivitis is usually accumulation of bacterial biofilms (plaque) [5]. Periodontitis is chronic multifactorial inflammatory disease of the periodontium characterised by progressive destruction of soft and hard tissues that support the teeth [6, 7]. The 2018 periodontitis classification scheme has grouped chronic periodontitis and aggressive periodontitis into a single category, periodontitis, which is defined as four stages based on severity and three grades (rate of progression, including grade modifiers smoking and diabetes mellitus) [6, 8].

The prevalence rates and severity of periodontitis in adults have been measured in population surveys. Mild and moderate forms of periodontitis (i.e. stages I and II) are prevalent in adults, with global prevalence rates reaching approximately 50% [9]. In 2010, severe periodontitis (stage III or stage IV) was the sixth most prevalent disease and was a major problem in 10.8% of people worldwide [4, 10]. Severe periodontitis increases in adults after age 30 years and is highest at age 40 years. Severe periodontitis appears to remain stable at older ages [4]. The age-standardised prevalence rate of severe periodontitis has increased from 1990 to 2019 [11]. Accordingly, one of the greatest challenges on oral healthcare is to decrease the prevalence of adult periodontal diseases. The latest results of the Finnish Health 2000 and 2011 surveys have remained unchanged; gingivitis was observed in 74% of adults, and 64% of adults had signs of periodontitis (i.e. stages I, II, III or IV) and 21% of adults had severe periodontitis (stage III or IV) [12].

Periodontitis is currently one of the primary causes of tooth loss in adults [8, 13]. One reason for periodontitis progression is that the early stage of disease is typically symptomless and patients cannot recognise the first signs of periodontal disease as gingival redness and occasional bleeding [10]. Pain is not a typical sign in the early stages of chronic gingivitis or periodontitis [10]. Periodontitis also has many risk factors based on lifestyle behaviours (such as smoking), poor oral hygiene, or non-modifiable variables (such as genetics). Diabetes is a significant risk factor for periodontitis and it can progress if an adult has poorly controlled or undiagnosed diabetes [14, 15]. Periodontal diseases are also associated with mental disorders such as depression, anxiety, or stress [16–20]. An association between cardiovascular disease and periodontitis was observed in those who reacted to an infection, with systematic inflammation [21, 22].

The focus of periodontal treatment (PT) is to stop the inflammatory process and to ensure the longevity and health of natural dentition [23]. However, it is challenging to predict progression from reversible gingivitis to irreversible periodontitis [24]. Therefore, supportive PT at appropriate intervals after the first PT can maintain periodontal stability in patients [10, 25, 26]. Unfortunately, patients with periodontal diseases often do not prioritise supportive PT even if they need it. Non-compliance rates with supportive after PT range from 28% to 75% [27–30].

Information on periodontal health status has been presented as the Community Periodontal Index (CPI) in epidemiological studies [31, 32]. The aim of this observational, follow-up, register-based study was to estimate the benefit of PT after oral health examination (OHE) based on follow-up OHE, using change in CPI as the periodontal health outcome in sextants.

## Materials and methods

This study was conducted in public oral health care clinics within the Helsinki City Social Services, Health Care and Rescue Services Division. All data were collected from registers. In total, 42,533 adults aged 18–89 years visited clinics between 01 January and 31 December 2009 and underwent an OHE. The individual recall interval (IRI) was between 0 and 60 months, and follow-up ended on 31 December 2015. Distribution of socioeconomic status (SES) in the study population was very similar to that of the general population of Helsinki in 2000 [33].

Until the end of November 2015, the process of accessing follow-up OHEs was the same as the primary OHEs in the year 2009, and no recall system was used for follow-up OHE. Both OHEs were defined as visits that included assessment of all oral tissues, a diagnosis, a treatment plan, and assignment of IRI. Information on oral health indices was obtained from computerised medical records of the visit when the IRI was determined [33]. Periodontal health was defined with CPI in primary and follow-up OHEs. Exclusion criteria were edentulism (*N* = 42), IRI under 12 months (as active treatment could be ongoing), or both. The final study population was 16,040 adults (Table 1).

**Table 1:** Basic characteristics of the study population (*N* = 16,040). Continuous variables: means (SD ^a^), categorical variables: frequencies (%).

Variables		Community Periodontal Index, maximum at first visit					
		Overall	0	1	2	3	4
*n*		16,040	739	1,097	10,359	3,024	821
Age, years, median (range)		44.80 [18.15, 92.60]	42.90 [18.80, 90.90]	40.59 [18.23, 90.97]	41.62 [18.15,92.60]	55.46 [18.69, 92.35]	60.96 [22.32, 92.36]
Sex, *n* (%)							
	Men	5,275 (32.9)	161 (21.8)	259 (23.6)	3,282 (31.7)	1,167 (38.6)	406 (49.5)
	Women	10,765 (67.1)	578 (78.2)	838 (76.4)	7,077 (68.3)	1,857 (61.4)	415 (50.5)
Socioeconomic status, *n* (%)							
	Self-employed or employer	353 (2.2)	19 (2.6)	22 (2.0)	247 (2.4)	55 (1.8)	10 (1.2)
		Community Periodontal Index, maximum at first visit					
		Overall	0	1	2	3	4
	Upper-level employee	2,614 (16.3)	144 (19.5)	190 (17.3)	1,951 (18.8)	295 (9.8)	34 (4.1)
	Lower-level employee	4,576 (28.5)	223 (30.2)	349 (31.8)	3,289 (31.8)	605 (20.0)	110 (13.4)
	Manual worker	1,898 (11.8)	70 (9.5)	129 (11.8)	1,270 (12.3)	360 (11.9)	69 (8.4)
	Student	533 (3.3)	36 (4.9)	34 (3.1)	393 (3.8)	60 (2.0)	10 (1.2)
	Pensioner	4,346 (27.1)	186 (25.2)	263 (24.0)	2,141 (20.7)	1,276 (42.2)	480 (58.5)
	Unemployed	1,162 (7.2)	49 (6.6)	76 (6.9)	695 (6.7)	259 (8.6)	83 (10.1)
	Unknown	558 (3.5)	12 (1.6)	34 (3.1)	373 (3.6)	114 (3.8)	25 (3.0)
Chronic disease, *n* (%)							
Cardiovascular disease, *n* (%)^b^	0	14,182 (88.4)	655 (88.6)	999 (91.1)	9,428 (91.0)	2,461 (81.4)	639 (77.8)
	1	1,858 (11.6)	84 (11.4)	98 (8.9)	931 (9.0)	563 (18.6)	182 (22.2)
Diabetes, *n* (%)	0	15 350 (95.7)	708 (95.8)	1,056 (96.3)	9,983 (96.4)	2,838 (93.8)	765 (93.2)
	1	690 (4.3)	31 (4.2)	41 (3.7)	376 (3.6)	186 (6.2)	56 (6.8)
		Community Periodontal Index, maximum at first visit					
		Overall	0	1	2	3	4
Severe mental disorder, *n* (%)	0	15,457 (96.4)	720 (97.4)	1,050 (95.7)	9,994 (96.5)	2,909 (96.2)	784 (95.5)
	1	583 (3.6)	19 (2.6)	47 (4.3)	365 (3.5)	115 (3.8)	37 (4.5)
DMFT, median (range)^c^		18.00 [0.00, 32.00]	17.00 [0.00, 32.00]	17.00 [0.00, 32.00]	16.00 [0.00, 32.00]	23.00 [0.00, 32.00]	24.00 [1.00, 32.00]
Number of teeth, median (range)		28.00 [2.00, 32.00]	28.00 [3.00, 32.00]	28.00 [2.00, 32.00]	28.00 [3.00, 32.00]	27.00 [2.00, 32.00]	25.00 [3.00, 32.00]
Length of follow-up/years, mean (SD)		2.56 (0.82)	2.64 (0.83)	2.55 (0.82)	2.63 (0.82)	2.39 (0.78)	2.24 (0.75)
Number of treatments in first year, *n* (%)	0	2,100 (13.1)	459 (62.1)	749 (68.3)	836 (8.1)	55 (1.8)	< 5 (0.1)
	1	7,164 (44.7)	118 (16.0)	153 (13.9)	5,696 (55.0)	1,080 (35.7)	117 (14.3)
	2	2,945 (18.4)	90 (12.2)	97 (8.8)	1,935 (18.7)	701 (23.2)	122 (14.9)
	3 or more	3,831 (23.9)	72 (9.7)	98 (8.9)	1,892 (18.3)	1,188 (39.3)	581 (70.8)
		Community Periodontal Index, maximum at first visit					
		Overall	0	1	2	3	4
Proportion with better CPI, mean (SD)		0.28 (0.33)	0.29 (0.37)	0.22 (0.33)	0.26 (0.33)	0.35 (0.31)	0.44 (0.31)
Number of extraction treatments, mean (SD)		0.42 (1.20)	0.31 (1.12)	0.29 (0.97)	0.31 (0.96)	0.61 (1.42)	1.46 (2.32)
IRI year 2009, *n* (%)^d^	[0, 12]	1,729 (10.8)	70 (9,5)	93 (8.5)	828 (8.0)	518 (17.1)	220 (26.8)
	(12, 24]	9,450 (58.9)	369 (49.9)	641 (58.4.)	6,100 (58.9)	1,876 (62.0)	464 (56.5)
	(24, 36]	4,290 (26.7)	263 (35.6)	324 (29.5)	2,987 (28.8)	583 (19.3)	133 (16.2)
	(36, 60]	571 (3.6)	37 (5.0)	39 (3.6)	444 (4.3)	47 (1.6)	< 5 (0.5)

Community Periodontal Index (CPI for the maximum value of an individual).

a SD: Standard deviation.

b Cardiovascular disease = chronic cardiac insufficiency, chronic hypertension, chronic coronary heart disease, and dyslipidaemia associated with coronary heart disease and chronic arrythmias.

DMFT: decayed, missing, filled teeth^c.^

IRI: individual recall interval in months^d.^

In Finland, there are specific procedural codes for OHEs and for treatments; these codes are provided and updated by the board of the Finnish Institute for Health and Welfare. All codes are combined with payments and treatments performed in public oral health clinics and in private clinics. Data from different sources can be combined through the computerised register using unique personal identification codes (PIC) of patients [34]. The following predictor variables were available: age, sex, oral health indices on date of OHE, SES, and information on chronic diseases. SES was divided into the following eight categories: self-employed or employer, upper-level employee, lower-level employee, manual worker, student, pensioner, unemployed, and unknown [33, 35].

As a proxy for chronic diseases, we used the special Drug Reimbursement Register of the Finnish Social Insurance Institution (SII) [36]. Drug reimbursement is based on a physician’s diagnosis and statements. We included the following diseases: diabetes mellitus, severe psychotic and other serious mental disorders (SK112; schizophrenia, psychosis affective, psychosis manodepressiva, paraphrenia involutionis, status paranoicus, paranoia, dementia senilis, dementia praesenilis, psychoses aliae), and cardiovascular diseases (CVD); such as chronic cardiac insufficiency, chronic hypertension, chronic coronary heart disease, and dyslipidaemia associated with coronary heart disease and chronic arrythmias, which were combined).

Following an OHE, patients who needed PT after OHE were given preventive care and non-surgical PTs by an oral healthcare team (dentist, hygienist, periodontist, or combinations thereof). Patients were referred to a periodontist in case of severe periodontitis due to the need for more demanding care (e.g. surgical PT).

The dentist recorded CPI for the full mouth. Dentition was divided into six sextants and health of the periodontium was explored at six sites per tooth. According to the CPI, the sextant should contain two functional teeth. The highest score for the component CPI for each sextant was recorded as follows: healthy (score 0), gingival bleeding on probing (score 1), calculus (score 2), periodontal pocket depth of 4–5 mm (score 3), and periodontal pocket depth ≥ 6 mm (score 4) [37]. The following explanatory variables were included in the analysis: age, sex, SES, number of treatments during PT, and non-modified diseases that are related to risk of periodontal diseases, such as CVD, diabetes, severe mental disorders and other serious mental disorders. We examined the influence of PT by sextants. Calculations were performed using R language [38].

We defined the ordinal scale outcome variable considering change between first OHE and follow-up OHE (determined by IRI) for each sextant. The outcome variable had three categories (worse, same, and better). Changes to the upper CPI value in the sextant were determined to be ‘worse’, changes to the lower value ‘better’, and unchanged value ‘same’. The outcome was defined as ‘better’ if both first and follow-up CPI score was 0. This ordinal scale outcome was modelled with proportional odds model for each sextant separately, and results were reported as odds ratios (ORs) with 95% confidence intervals (CIs) [39]. We also modelled the proportion of better sextants with logistic regression using quasi-likelihood that takes over-dispersion into account. Results of logistic regression are also reported as ORs with 95% CIs.

The study protocol was approved by the Ethics Committee of the Faculty of Medicine at the University of Helsinki (08 September 2017, reference 09/2017). Permits to use the register data were obtained from the City of Helsinki (05 January 2018, reference 2017-013665), Statistics Finland (03 January 2019, reference TK-52-41-19, TK/2571/07.03.00/2022, 30 September 2022, FinData Dnro THL/3500/14.06.00/2022), and the SII (31 January 2019, reference 9/522/2019, 30 September 2022, FinData Dnro THL/3500/14.06.00/2022).

## Results

The study population (*N* = 16,040) consisted of 10,765 women and 5,275 men. Baseline characteristics and prevalence of periodontal diseases (CPI) are summarised in Table 1. Mean follow-up time of OHEs was between 2.2 and 2.6 years, and was shortest in patients with CPI score 4. A healthy periodontium was recorded for 4.6% (*N* = 739) of patients and periodontal deep pockets (CPI scores 3 or 4) were found in 24% (*N* = 3,845) of patients (Table 1). The highest mean age of 61.0 (22.3, 92.4) years was observed in CPI score 4; the number of teeth were lowest in the same group. Nearly all patients (91%) had teeth in six sextants (Supplementary Table 1). The most prevalent score was 2 (presence of calculus) in all sextants in OHEs and in follow-up OHEs (Supplementary Table 2).

**Figure 1:**
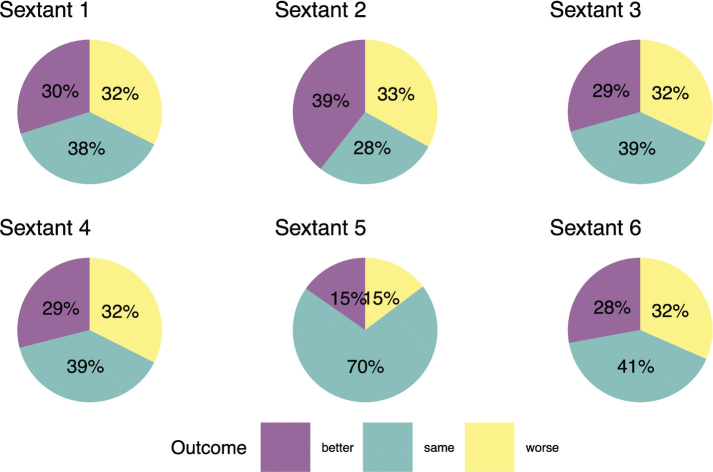
Changes of Community Periodontal Index between baseline and follow-up visits by different sextants in dentition.

Treatment codes indicated that if CPI score was ≥ 1, the patient should have one PT or more depending on severity of gingivitis or signs of periodontitis. In the study population (*N* = 1,645), 10% of patients deemed to need PT did not receive it. Seven out of ten of these patients had CPI score 1 or 2. The distribution of periodontal health by different sextants in the follow-up OHE indicated that the best response to treatment was in sextant 2 (Figure 1). A total of 56% of patients had at least one better sextant in follow-up OHE and all sextants were better for 7% (Supplementary Table 1). During PT, patients with CPI score 4 had more teeth extractions than patients with other CPI scores (Table 1).

**Figure 2:**
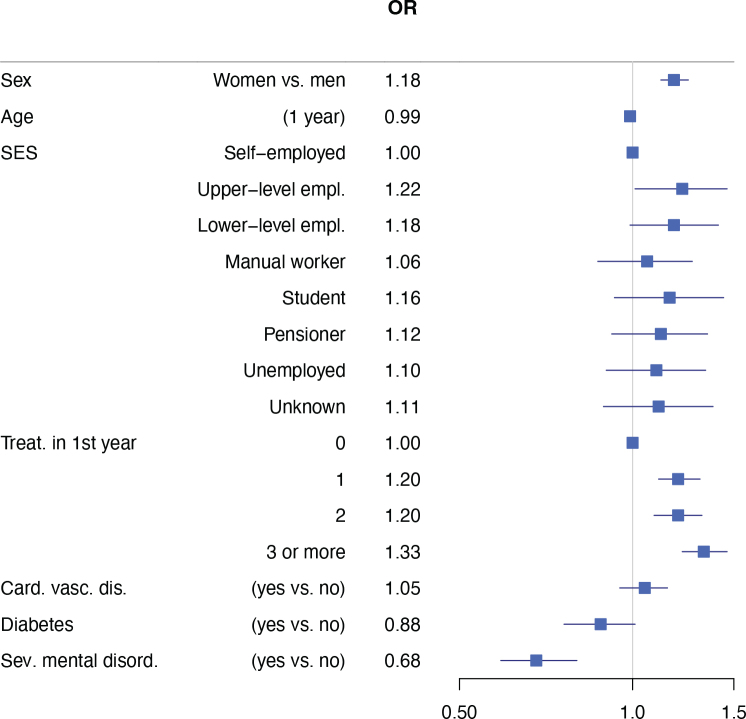
Odds ratios and 95% confidence intervals for proportion of better CPI. Results based on quasi binomial models for all sextants combined. Cardiovascular disease = chronic cardiac insufficiency, chronic hypertension, chronic coronary heart disease, and dyslipidaemia associated with coronary heart disease and chronic arrythmias.

The results of models indicated improvement of CPI of sextants if periodontal diseases were managed with PT by an oral healthcare team (Table 2). There was no significant interaction between PT and chronic diseases. However, diabetes or severe mental disorders were associated with poorer outcome to PT in our models in all sextants within the follow-up OHE (Table 2) or in combined sextants (Figure 2). In addition, men had poorer outcome to PT than women in the model in which all sextants were combined (Figure 2, Supplementary Table 3).

**Table 2:** Odds ratios and 95% confidence intervals for better Community Periodontal Index value based on proportional odds models, separately for sextants.

Variables		Odds ratio with 95% confidence intervals					
		Sextant 1	Sextant 2	Sextant 3	Sextant 4	Sextant 5	Sextant 6
**Sex**	Women vs. men	1.08 (1.01–1.15)	1.16 (1.09–1.23)	1.06 (0.99–1.13)	1.05 (0.99–1.12)	1.04 (0.97–1.12)	1.03 (0.96–1.10)
**Age**	(1 year)	0.99 (0.99–1.00)	0.99 (0.99–0.99)	0.99 (0.99–1.00)	0.99 (0.99–1.00)	0.99 (0.99–1.00)	0.99 (0.99–1.00)
**Socioeconomic status**	Self-employed	(reference)	(reference)	(reference)	(reference)	(reference)	(reference)
	Upper-level employee	1.04 (0.85–1.28)	1.27 (1.03–1.56)	1.22 (1.00–1.50)	1.10 (0.90–1.35)	1.17 (0.92–1.48)	1.01 (0.82–1.25)
	Lower-level employee	1.05 (0.86–1.28)	1.23 (1.00–1.50)	1.21 (0.99–1.47)	1.11 (0.91–1.36)	1.16 (0.92–1.47)	1.06 (0.87–1.30)
	Manual worker	0.96 (0.77–1.18)	1.13 (0.91–1.39)	1.08 (0.88–1.33)	1.06 (0.86–1.31)	1.07 (0.84–1.37)	0.98 (0.79–1.21)
	Student	0.91 (0.70–1.17)	1.16 (0.90–1.49)	1.09 (0.85–1.40)	1.06 (0.83–1.37)	1.21 (0.90–1.61)	1.02 (0.80–1.32)
	Pensioner	0.98 (0.79–1.21)	1.22 (0.99–1.50)	1.13 (0.92–1.40)	1.02 (0.83–1.26)	1.09 (0.86–1.40)	0.95 (0.77–1.17)
	Unemployed	1.01 (0.81–1.27)	1.15 (0.92–1.43)	1.25 (1.01–1.56)	1.09 (0.87–1.35)	1.13 (0.87–1.46)	1.01 (0.81–1.26)
	Unknown	1.05 (0.82–1.35)	1.17 (0.91–1.49)	1.26 (0.98–1.61)	1.07 (0.84–1.38)	1.19 (0.89–1.58)	1.07 (0.83–1.37)
**Treatments in first year**	0	(reference)	(reference)	(reference)	(reference)	(reference)	(reference)
	1	2.51 (2.27–2.77)	1.39 (1.26–1.53)	2.50 (2.27–2.77)	2.51 (2.27–2.77)	5.05 (4.53–5.63)	2.62 (2.38–2.90)
	2	2.59 (2.31–2.89)	1.41 (1.26–1.57)	2.47 (2.21–2.77)	2.55 (2.28–2.86)	4.54 (4.00–5.14)	2.55 (2.28–2.85)
	3 or more	2.73 (2.45–3.05)	1.41 (1.27–1.57)	2.58 (2.31–2.89)	2.78 (2.49–3.10)	5.24 (4.64–5.92)	2.94 (2.63–3.28)
**Cardiovascular disease** ^a^	(yes vs. no)	1.01 (0.91–1.12)	1.02 (0.92–1.13)	1.09 (0.98–1.21)	1.03 (0.93–1.14)	1.02 (0.90–1.15)	1.03 (0.92–1.14)
**Diabetes**	(yes vs. no)	0.86 (0.73–1.00)	0.90 (0.78–1.04)	0.88 (0.75–1.03)	0.97 (0.83–1.12)	0.92 (0.77–1.10)	0.92 (0.79–1.07)
**Severe mental disorder**	(yes vs. no)	0.81 (0.69–0.95)	0.75 (0.64–0.88)	0.81 (0.69–0.95)	0.84 (0.71–0.98)	0.87 (0.72–1.05)	0.90 (0.77–1.05)

a Cardiovascular disease = chronic cardiac insufficiency, chronic hypertension, chronic coronary heart disease, and dyslipidaemia associated with coronary heart disease and chronic arrythmias.

## Discussion

This observational, register-based, follow-up study provides information on outcome of PTs in follow-up of OHE in real-life conditions. The main result was that PT after OHE is important for better periodontal health of adults. In model of combined sextants, we observed better outcome with higher number of treatments. Odds ratio for better CPI for one or two treatments was 1.2, and for three or more 1.3. However, outcome was poorer for patients with diabetes or severe mental disorders, and for men. The lack of an interaction showed that chronic diseases do not modify the effect of PT.

Substantial evidence indicates that periodontitis increases systematic low-grade inflammation [40, 41]. The objective of PT is to reduce this inflammation [41]. To date, treatment of gingivitis has been a key component in preventing periodontitis [23]. The importance of prompt PT, including oral hygiene instruction, based on OHE and diagnosis is the first step towards better periodontal health of adults and should be highlighted. However, removal of subgingival biofilms need frequent treatment by professionals [42, 43]. Thus, all patients with signs of periodontal diseases, with or without risk factors (such as smoking, uncontrolled diabetes, and poor oral hygiene with plaque accumulation and calculus) should be referred to PT immediately after OHE.

In this study, the CPI of sextant was used to measure change in periodontal status based on clinical examinations. The most prevalent CPI was score 2 in all sextants in both OHEs; these findings support the results of previous studies [31, 44]. The results of follow-up OHEs confirmed earlier findings of recurrence of calculus [45]. Removal of supragingival and subgingival biofilm and rough calculus from diseased root surfaces is performed to prevent periodontal inflammation and periodontal diseases, such as periodontitis [46, 47].

A priority of PT is to improve patient adherence with better personal oral hygiene in the future [48, 49]. In this study, information of PT was based on nonsurgical treatment codes, which describes treatments as preventive treatments, scaling, and root planing. PT was the first active treatment period after OHE and the efficacy of PT can be described as lack of inflammatory signs or better CPI score of follow-up OHE. The effect of non-surgical PT depends on pocket depth, tooth type, and tooth surface [46, 50]. The CPI results of this study indicate that treatment response varies by sextants in follow-up OHEs. Periodontal health in follow-up was better in sextant 2 (Figure 1). However, the models treatment response was the best in the sextant 5. Response of treatment in sextant 5 may be explained by anatomical factors and tooth morphology [51]. A retrospective 3-year longitudinal evaluation of periodontal therapy using CPITN index (currently CPI) revealed that treatment outcome for anterior sextants was equivalent or better than in posterior sextants [37]. Although 24% of posterior sextants with CPI score 3 and 62% with CPI score 4 were treated surgically, almost all anterior sextants were treated non-surgically [37]. The significance of tooth morphology and periodontium health was shown in a register-based cohort study of furcation status in mortality of molars, which demonstrated that furcations have a strong association with molar loss [52]. Longitudinal studies of PT of multirooted teeth showed that typically multirooted teeth with furcation needs surgical treatment more often than non-furcation teeth [53]. In contrast, initial (degree 1) furcation could be treated non-surgically [53].

The appropriate treatment plan for periodontitis is complicated by many associated risk factors, not least various systematic diseases. Periodontitis is associated with a high risk for CVD [21, 54–56], and poor treatment response may increase the risk of future CVD events [56, 57]. Diabetes affects over 8% of the world population [51], and is one of the most important risk factors for periodontitis [51]. Furthermore, individuals with diabetes have increased risk for severe periodontitis [58]. Diabetes and periodontitis are linked in a two-way association; uncontrolled diabetes is a major risk factor for periodontitis [31, 58], and severe periodontitis influences glycaemic control in people with diabetes or at risk for diabetes. Stress and different mental disorders are currently addressed as risk factors for periodontal diseases, especially periodontitis [17, 18, 59]. However, several studies have shown conflicting results between mental health and periodontal health. In these studies, information on mental health was based on self-reported questionnaires [60, 61] and different study protocols preclude comparison of results.

In our study, the mental health of patients was categorised according to the International Classification of Diseases, and we found a statistically significant association between mental health and periodontal diseases. A healthy periodontium (CPI score 0) was observed only in 3% of patients with mental disorders, while the proportion was greater in other risk groups (4% and 11% in patients with diabetes and CVD, respectively). The poor outcomes in sextant models of PT in patients with severe mental disorders were consistent with the early study of Elter et al. [59].

Our results from models showed improvement of CPI of sextants based on number of treatments. A similar result was observed if the model was based on combination of sextants. The risk of periodontal diseases is higher for men than women [9, 17]. In this study, men were at risk for poorer periodontal health than women after PT in follow-up OHEs. A longitudinal study revealed that gingivitis is a risk factor for periodontitis and even for tooth loss in men [62]. Therefore, the oral healthcare team should ensure that men commit to PT, adequate oral hygiene, and also supportive PT if needed.

Gingivitis and periodontitis can be easily diagnosed and successfully treated and controlled following appropriate professional care by an oral healthcare team [10]. The results of this study also emphasise the need for multiprofessional competence to provide better overall care (i.e. medical care with the oral healthcare team). The decision of supportive PT after PT should be based on need and general health of patients [17]. Systematic diseases, such as diabetes and severe mental disorders, likely affect host response and make individuals more susceptible to disease [51, 59, 63, 64]. Individuals with diabetes or severe mental disorders have poor wound healing, which is relevant to PT [63, 65]. Periodontium health is important for adults with diabetes, as poor periodontal health increases the risk of development of systemic complications [14, 58]. On the other hand, poor stress tolerance may increase the risk of different diseases, such as periodontitis [66]. Individuals with severe mental disorders may have behaviours such as poor oral hygiene or smoking, which affect the periodontium and are related to periodontitis [67]. Antidepressants can cause xerostomia, which can influence periodontal health [59]. Our results suggest that individuals with diabetes or severe mental disorders need different treatment pathways than those without these diseases, and men in particular should be supported to receive PT as appropriate.

Our study is not without limitations. As this was a register-based study, we did not obtain information about tobacco use, diet, alcohol intake, oral hygiene habits, or X-ray findings from patient records. We did not have information on diabetic patients’ HbA1c levels to assess degree of disease control. Furthermore, it was not possible to extract specific details on preventive treatments, as these were included under an umbrella PT code. CPI as a measure for PT may be considered problematic, as it was originally designed for screening PT in the population. However, CPI nevertheless reflects the seriousness of periodontitis and in our opinion is useful in large epidemiological studies such as the present study. The definition of our outcome variable also has limitations, as it does not consider the degree of change in CPI but only indicates direction (‘worse’, ‘same’, ‘better’). However, we argue that in a large study this is sufficiently robust to be useful. It is always possible that in observational study there is confounding or other types of bias. However, we attempted to mitigate bias with statistical modelling.

A strength of our study is that it is population-based and is representative of the adult population of the City of Helsinki, and thus we have large individual levels of follow-up data [33]. Another strength of our study is that information on PTs was based on national codes used in everyday clinical practice. The indices of oral health, with potential confounding factors, such as SES and chronic diseases based on PIC, allowed us to link data from different registers. Finally, by adjusting all variables, we observed a link in PT outcome between OHEs, follow-up OHEs, and patient’ general health.

## Conclusions

Maintaining a healthy periodontium requires patient’ commitment to PT, better oral hygiene habits like frequent tooth brushing twice a day and interdental cleaning [43], and healthy lifestyle decisions, such as quitting smoking. Individuals with signs of periodontal diseases should identified and also evaluated for the risks for future progression of periodontitis by an oral healthcare team.

Health of adults should be evaluated at every OHE because of the risk of undiagnosed or uncontrolled diabetes affecting periodontal health. Our findings support the importance of the dentist informing patients about the benefits of PT, follow-up OHE, and supportive PT for those who need it. Besides diabetes, severe psychotic and other serious mental disorders should be considered as risk factors for periodontitis. PT should start immediately after OHE by the entire professional oral healthcare team, including specialists, according to patient needs. Although our data from Helsinki City have their unique characteristics, we argue that results of our study may be generalised in caution to most European populations.

## Supplementary Material

Effects of periodontal treatment on periodontal status in Finland: a register-based study

## Data Availability

The data that support the findings of this study are available from Statistics Finland, the SII, Social Services, Health Care and Rescue Services Division City of Helsinki. Restrictions apply to the availability of these data, which were used under license for the current study, and thus are not publicly available. However, data are available from the authors upon reasonable request and with the permission of Statistics Finland, the SII, Social Services, Health Care and Rescue Services Division City of Helsinki.
