# Supplementary material for: Effects of periodontal treatment on periodontal status in Finland: a register-based study
Source: Acta Odontol Scand. 2025 Mar 18;84:43232. doi: 10.2340/aos.v84.43232 (PMC11971947; doi:10.2340/aos.v84.43232)
Supplement: Effects of periodontal treatment on periodontal status in Finland: a register-based study [file AOS-84-43232-s1.pdf]

Supplementary material has been published as submitted. It has not been copyedited or typeset by Acta Odontologica Scandinavica.

Supplementary Table 1. Rows number of sextants per individual, columns number of sextants with better outcome.

	0	1	2	3	4	5	6	Sum
**1**	97	35	0	0	0	0	0	132
**2**	95	33	22	0	0	0	0	150
**3**	161	53	23	26	0	0	0	263
**4**	155	68	34	29	21	0	0	307
**5**	257	106	74	41	37	38	0	553
**6**	6286	2454	1436	1258	921	1343	937	14 635
**Sum**	7051	2749	1589	1354	979	1381	937	16 040

Supplementary Table 2. CPI (Community Periodontal Index) of sextants per individual based on baseline (row) and follow-up oral health examination (column).

CPI	Level	Overall	0	1	2	3	4	X
Sextant 1 (%)	0	2583 (16.1)	628 (30.5)	706 (22.0)	1038 (14.4)	169 (7.4)	22 (5.5)	20 (2.3)
	1	3043 (19.0)	499 (24.2)	891 (27.8)	1345 (18.7)	254 (11.1)	27 (6.7)	27 (3.1)
	2	7249 (45.2)	800 (38.8)	1359 (42.4)	3990 (55.4)	900 (39.3)	105 (26.1)	95 (10.8)
	3	2033 (12.7)	105 (5.1)	199 (6.2)	720 (10.0)	789 (34.5)	146 (36.2)	74 (8.4)
	4	377 (2.4)	13 (0.6)	35 (1.1)	56 (0.8)	152 (6.6)	92 (22.8)	29 (3.3)
	X	755 (4.7)	16 (0.8)	17 (0.5)	55 (0.8)	25 (1.1)	11 (2.7)	631 (72.0)
Sextant 2 (%)	0	5106 (31.8)	2114 (48.8)	1435 (34.6)	1412 (24.1)	118 (11.0)	15 (8.6)	12 (2.6)
	1	3928 (24.5)	1096 (25.3)	1269 (30.6)	1374 (23.4)	157 (14.7)	17 (9.8)	15 (3.3)
	2	5591 (34.9)	1039 (24.0)	1313 (31.6)	2718 (46.4)	442 (41.3)	46 (26.4)	33 (7.2)
	3	853 (5.3)	65 (1.5)	113 (2.7)	314 (5.4)	282 (26.4)	51 (29.3)	28 (6.1)
	4	185 (1.2)	10 (0.2)	16 (0.4)	35 (0.6)	64 (6.0)	44 (25.3)	16 (3.5)
	X	377 (2.4)	6 (0.1)	3 (0.1)	8 (0.1)	7 (0.7)	1 (0.6)	352 (77.2)
Sextant 3 (%)	0	2470 (15.4)	590 (28.7)	632 (21.6)	1038 (13.7)	166 (7.5)	16 (4.1)	28 (3.1)
	1	2875 (17.9)	471 (22.9)	763 (26.1)	1354 (17.9)	236 (10.7)	16 (4.1)	35 (3.9)
	2	7559 (47.1)	861 (41.9)	1285 (43.9)	4292 (56.8)	900 (40.8)	113 (29.2)	108 (11.9)
	3	1997 (12.5)	99 (4.8)	194 (6.6)	751 (9.9)	741 (33.6)	134 (34.6)	78 (8.6)
	4	360 (2.2)	16 (0.8)	22 (0.8)	70 (0.9)	127 (5.8)	99 (25.6)	26 (2.9)
	X	779 (4.9)	19 (0.9)	31 (1.1)	53 (0.7)	35 (1.6)	9 (2.3)	632 (69.7)
Sextant 4 (%)	0	2695 (16.8)	644 (31.4)	685 (21.4)	1175 (14.7)	136 (7.5)	19 (7.3)	36 (4.9)
	1	3096 (19.3)	505 (24.6)	877 (27.4)	1434 (18.0)	214 (11.9)	18 (6.9)	48 (6.5)
	2	7864 (49.0)	795 (38.7)	1447 (45.1)	4526 (56.7)	870 (48.2)	83 (31.9)	143 (19.3)
	3	1514 (9.4)	80 (3.9)	148 (4.6)	679 (8.5)	485 (26.9)	84 (32.3)	38 (5.1)
	4	220 (1.4)	10 (0.5)	7 (0.2)	64 (0.8)	78 (4.3)	53 (20.4)	8 (1.1)
	X	651 (4.1)	18 (0.9)	41 (1.3)	99 (1.2)	21 (1.2)	3 (1.2)	469 (63.2)
Sextant 5 (%)	0	1388 (8.7)	278 (22.8)	180 (16.8)	897 (7.1)	27 (3.2)	3 (2.1)	3 (4.4)
	1	882 (5.5)	129 (10.6)	166 (15.5)	557 (4.4)	26 (3.1)	2 (1.4)	2 (2.9)
	2	12 884 (80.3)	788 (64.7)	697 (65.0)	10 789 (84.9)	533 (64.1)	62 (43.4)	15 (22.1)
	3	696 (4.3)	16 (1.3)	27 (2.5)	410 (3.2)	200 (24.0)	32 (22.4)	11 (16.2)
	4	145 (0.9)	5 (0.4)	1 (0.1)	48 (0.4)	44 (5.3)	43 (30.1)	4 (5.9)
	X	45 (0.3)	2 (0.2)	2 (0.2)	5 (0.0)	2 (0.2)	1 (0.7)	33 (48.5)
Sextant 6 (%)	0	2439 (15.2)	565 (29.8)	569 (18.9)	1112 (13.4)	148 (8.0)	9 (3.2)	36 (5.0)
	1	3019 (18.8)	454 (24.0)	858 (28.5)	1438 (17.4)	205 (11.1)	17 (6.0)	47 (6.5)
	2	8162 (50.9)	779 (41.2)	1394 (46.3)	4850 (58.6)	909 (49.0)	89 (31.4)	141 (19.5)

	3	1553 (9.7)	70 (3.7)	144 (4.8)	716 (8.6)	483 (26.0)	98 (34.6)	42 (5.8)
	4	258 (1.6)	13 (0.7)	10 (0.3)	76 (0.9)	80 (4.3)	65 (23.0)	14 (1.9)
	X	609 (3.8)	12 (0.6)	34 (1.1)	86 (1.0)	30 (1.6)	5 (1.8)	442 (61.2)

Table 3. Odds ratios (ORs) and 95% confidence intervals for proportion of better CPI.

		OR
**Sex**	Women vs. men	1.18 (1.12–1.25)
**Age**	(1 year)	0.99 (0.99–1.00)
**Socioeconomic status**	Self-employed	(reference)
	Upper-level employee	1.22 (1.01–1.46)
	Lower-level employee	1.18 (0.99–1.41)
	Manual worker	1.06 (0.87–1.27)
	Student	1.16 (0.93–1.44)
	Pensioner	1.12 (0.92–1.35)
	Unemployed	1.10 (0.90–1.34)
	Unknown	1.11 (0.89–1.38)
**Number of treatments in first year**	0	(reference)
	1	1.20 (1.11 - 1.31)
	2	1.20 (1.09 - 1.32)
	3 or more	1.33 (1.22 - 1.46)
**Cardiovascular disease** [a]	(yes vs. no)	1.05 (0.95-1.15)
**Diabetes**	(yes vs. no)	0.88 (0.76 - 1.01)
**Severe mental disorder**	(yes vs. no)	0.68 (0.59 - 0.80)

Value based on quasi binomial models for all sextants combined.

Cardiovascular disease = chronic cardiac insufficiency, chronic hypertension, chronic coronary heart disease, and dyslipidaemia associated with coronary heart disease and chronic arrythmias [a]
